# On Some Phenomena of Vision

**Published:** 1818-05

**Authors:** Nich. Littleton


*For the London Medical and Physical Journal.*

### *On some Phenomena of Vision;* by Nich. Littleton, Esq.

WITH much pleasure I have read Dr. Adams's letter on his own interesting case; in which he accounts differently for the phenomena which occured, than had been before done by Mr. Ware.

It is mentioned, that Mr. Stevenson saw something *hanging from the iris.* If such was the cause of the fly-like appearance, such appearance would be, perhaps, rather more distinct in much than in little light; as, in the diminished state of the pupil, the opaque body must bear a greater proportion to the whole area of the pupil than in the enlarged state. At least, this would be the case where the opaque body hangs from the very edge of the iris. If, however, it should hang from the iris, nearer to its peripheral annulus, it might not be possible to see it during the diminished state of the pupil; when, of course, it could not cause the appearance of muscæ. But, let this opaque body hang from any part of the iris, still the phenomena consequent on exposing the eye to different degrees of light may differ from such as would arise, where the fault was in the retina. Because, in an affection of this last-mentioned part, the appearance of muscæ is most distinct in a bright light, and sometimes cannot be observed without it; whereas, in either of the former cases, it must always be seen plainly whenever the pupil is enlarged,—whenever the iris is exposed to but little light.

Dr. Adams has not mentioned the effect of different degrees of light, but it may be still within his recollection.

Dr. A. remarks, that the appearance remained, although the eyelids were closed. Now, I would ask, how this can be accounted for by adopting his opinion?—I should as soon expect that persons would be sensible of specks on their cornea, when the eyes are shut. This continuance of the appearance, when the lids are closed, can, however, be so readily accounted for, from pressure on the retina, that it is unnecessary to add any explanation.

Dr. Adams lays much stress on the determined outline; but, I would ask, whether it is not more likely for a single body to appear of a more determinate shape, since the attention is less diverted, than where the retina receives numerous impressions,—where many muscæ volitantes appear, as in the cases mentioned by Mr. Ware?

Two of Mr. W.'s patients remarked, that the muscæ appeared larger when the eye was directed to distant objects than when near objects were viewed. Now, adopting Dr. A.'s opinion, this is readily accounted for, from the very same principle that we often fancy small objects to be of a large size, when they are near to the eye, at the time that the eye is directed to a distant object. Thus, I have often, for an instant, thought that I saw something of a large size which I have soon discovered to have been only a fly; but, pressure on the retina equally accounts for this. Our judgment of the size of objects, arises from experience and habit; and a fly, close to the eye, would cause as large an image on the retina, as would an ox at a distance; also, an area on the retina, of the same size, would be adjudged to arise from the impression of a small or large body, according as we thought the object to be near or far off. For, whenever the mind cannot judge of distance, neither can it judge of magnitude.

The cases mentioned by Mr. W. differ from that of Dr. A. in being preceded by, or accompanied with, other irregularities of vision. And, although in Dr. A.'s own case, his opinion concerning its cause should be adopted, nevertheless, such is not likely to be the cause of phenomena so different as in Mr. W.'s cases, especially considering their duration.

It is true, that I am not accustomed to the use of microscopes; but, from the discrepancy of those who are or have been, from the little experience I have had, and from my reasonings as to its utility in many cases to which it is or has been applied, I am not inclined to attend much to microscopical observations in the present case: as it seems to be hardly possible thus to observe, with accuracy, any object that could so interfere with vision, which could not also be seen with the naked eye. It does not also seem very possible to well apply a microscope, in order to observe the interior of the eye, as one must look rather obliquely through the cornea, in order to avoid the images otherwise reflected, and thus obstructing the view. I have also sometimes thought that I saw something of a colour a little different from the iris, just behind its edge; of which I was still doubtful, as, perhaps, it might have been merely the reflected image of the ciliæ or tarsus of the eye viewed. Dr. Adams also seems not to have been convinced, that Mr. Stevenson's observation was entirely free from deception; as he supposes, that the spectrum was caused by a portion of the pigmentum *fallen on the lens;*—a situation in which it must be very unlikely for one to observe bodies, unless very much differing in colour from blackness, as all the light, by which it could be discerned, must be admitted in the same direction in which such bodies are observed.

These are reasons why I should be inclined, with Mr. Ware, to consider muscæ volitantes as an affection of the retina. This affection of the retina seems also very likely to arise from the pressure caused by a distended, or even varicous, state of the vessels of the choroïd. As these distensions may be only here and there, or even in a single spot, it may be accounted for, why many or a single object alone is seen. And, as the pressure from such distension may differ in degree, it likewise accounts for those objects being, in some cases, seen only when the eye is exposed to much light; whilst, in other cases, the patient may be sensible of their presence, although the lids are closed; and, in the greatest degree of pressure, portions of the retina may be rendered altogether insensible.

Thus, considering muscæ volitantes to arise from the pressure of vascular distension, such appearances would be more distinct, whilst more blood was determined to the head, as during suspensions of respiration, &c.—*Question*, Did this occur in Dr. Adams's case?

Mr. Ware does not seem determined in considering the cases he mentions as nervous, neither does he define his meaning of nervous. He seems only to intend to show, that the appearance of the muscæ are not dangerous to vision, and only an inconvenience. Now, all this may be allowed, although it be considered as arising from vascular distension. M. H. has had a sensation of beating and whizzing in the right side of her head for twelve years, with little variation. It ceased on compressing the carotids. It is hardly necessary to state this, as, without doubt, every medical gentleman recollects such cases. Thus, proving that determination of blood to particular parts may continue for a long time, without destroying the function of the part, and with only trifling inconvenience.

One may cause somewhat the appearance of muscæ by an universal pressure on the globe, as with the palm of the hand, especially that sort mentioned in Mr. W.'s first case, where bright objects, like globules of mercury, were seen. Pressure, however, so applied, cannot but be more extensive; the appearance of objects excited must, therefore, appear throughout the whole sphere of vision. I am inclined to think, that the least degrees of pressure cause the spectra to appear coloured, and that increased pressure causes them to appear black. The following experiment of Dr. Porterfield induces the supposition. Let the globe o the eye be slightly pressed on with the tip of the finger, near to either canthus, but especially on the superior part of the inner one: the force thus applied causes pressure on the retina, hence towards the opposite part of the globe, (the direction in which light must fall in order to strike on this part of the retina,) there will be an appearance of a circular dark spot, surrounded with an annulus of a bright colour. Now, this brighter colour at the circumference is easily accounted for from the less degree of pressure which the retina suffers at that part, since it is impossible to compress the retina to such an extent as to cause dark-coloured spectra, without exposing a surrounding annulus of its surface to a less pressure, because of the interposition of the sclerotic coat. Here, also, I may remark, the very slight pressure causing the sensation of light. A degree of pressure, scarcely amounting to a slight touch on the lid at the superior part of the inner canthus, is enough to cause an appearance of slight brightness in the same situation as the dark spectrum, caused by greater pressure, appears. This appearance of brightness, without the centre of darkness, is so slight, that it is scarcely perceptible, and would never be observed but by those who are familiarized with the dark appearance. It is extremely probable, that a still less degree of pressure would cause spectra, when affecting the more sensible part of the retina; and such spectra might, perhaps, be infinitely more luminous. Few there are, who have not, from blows on the eye, seen fire.

I have mentioned, that a varicous state of the choroïd might be to such an extent as to cause even insensibility in some parts of the retina, and that without affecting the sensibility of other parts of it. I have not had an opportunity of having the most certain proof of such a varicous state, viz. the evidence arising from dissection. Such an examination, however, might not afford proof so certain as I might expect, since such slight pressure is sufficient to cause insensibility. My evidence hinges on the phenomena observed in the following case, and on consequent deductions.

About six months since, I saw Mrs. Yendall, æt. 40, of a strong firm fibre. She lives in the village of Landrake, in the eastern part of the county. From herself and friends I got the following information:—That, about sixteen months before this time, she was seized with convulsion, being then about seven months advanced in her first pregnancy. She was delivered within, I think, about twenty-four hours after. Whilst convulsed, bleeding was attempted, but no blood flowed. She continued convulsed for two or three days, and remained in a state of insensibility for about three weeks, at which time she noticed her husband whilst standing at the foot of the bed. After this time, she gradually recovered her lost faculties. It was a considerable time before her memory was equal to the counting the striking of the clock, and now she cannot remember the text of a sermon, notwithstanding she remembers better than her friends any thing of importance to her complaint. She now seems tolerably well, and all her senses, but that of seeing, are perfect. There is not, nor has not been, any strabismus, neither is there any unnatural appearance of the eyes; the irides sympathising, and being affected, as are healthy eyes. She occasionally complains of head-ach, but by no means severe. By examining her, I found she could see the smallest print at different distances with either eye; but she has not the power of judging her distance, or even of the direction in which objects around her are placed. Putting a book on the table, within her reach, I desired her to put her hand to it repeatedly: I found that she directed her hand to a greater or less distance from it, sometimes to the right, sometimes to the left, sometimes on this side of it, sometimes beyond it, and sometimes even in a direction almost contrary to it. To be short,—she found it as one blind would, rather than by any power of vision, first feeling for the edge of the table, and then here and there on the table, although she said she plainly saw it all the time. I desired her to try to put her hands to many other things in various directions, sometimes allowing both eyes to be open, sometimes shutting the right or left eye,—but no difference.

If her hand was directed by any one to an object which she saw, she could directly after withdraw and reapply her hand to it repeatedly, whilst she and the object were unmoved; showing that her muscular habits were superior to her deceitful vision, the judgment not allowing her vision to mislead such habits. I found that she could see, and had discovered, for some time, her capability of seeing the hours I. II. V. VI. IX. X. XI. marked on the dial-plate of a common clock, but she could not see the other hours. This certainly proved that only small portions of the retina were sensible, and that other parts were insensible. She had not, it was evident, a spot sensible in either eye sufficiently large to receive the impressions of three or four characters of the dial. I then tried her with the print of a Common Prayer-book, when I found that she could see short words, but was unable to see nine or ten letters together. On desiring her to read the Lord's Prayer, she would, after moving her head about for some time, perhaps catch the first word of the prayer, and then another word some way on in the line, or, perhaps, several lines below; and never could discern any two words, each containing eight or nine letters, in succession to each other. Thinking that one eye might confuse the other, I tried each eye separately, but without any advantage. From her manner of discovering objects by moving her head and eyes, which she did for a considerable time before she saw any short word she might be desired to look at; I conclude that there is but a very small portion of the retina remaining sensible, perhaps, only a single spot in each eye; sometimes she could not discover even a small word, although she was told that it was just above, or just below, or on the right or left of, any other word which she could see. One of her relatives, who was present, seemed to give a full account of her state of vision in very few words,—remarking to me that her daughter-in-law had not lost her sight, but only the *guide of her sight.* From thus observing that she could only see small portions of surface, her inability of judging of the distance and direction of objects around her was accounted for. In order to be able to direct the hand to an object, it is not merely necessary that we should see such objects, but we must also be capable of seeing the hand, or other part of our body, or any other substance which we would direct towards it, and likewise the space between the object and the hand, or other substance. This seems evidently necessary to one reflecting for an instant, although it has not been remarked by any one heretofore that I know of, because, perhaps, such an occasion for remarking it had not been before observed. Former writers have properly remarked, that the angle in which the rays fall on the retina, the crossing of the optical axis of two eyes, together with experience and habit, enable us to judge of distance, &c.; but it now seems plain, that these are not alone sufficient. As this female is unable to see the hand, the object, and the intermediate space, at the same time, but only one of

2

these at once, she is, therefore, rendered incapable of judging of the distance and direction of surrounding objects. From all these corresponding phenomena, it seems evident, that the greater portion of the retina was insensible, and yet a small portion of it remained perfectly sensible. This imperfection was consequent to an attack of violent convulsion, which acted, undoubtedly, by causing a congestion of blood in the vessels of the eyes, and most probably a varicose state of the vessels of the choroïd throughout the greater part of that membrane which is situated behind that portion of the retina which receives the impression of external objects.

These are all the phenomena which I remarked on a single visit; now, however, other questions suggest themselves, as —Does she see objects with one or both eyes at once? &c. If, therefore, at a future visit, other facts worth recording are detected, I hope such will be publicly made known.

During and after her convulsion, she was attended by two surgeons of the neighbourhood; and since, she told me, she had applied to a surgeon at Plymouth Dock, with the hope that electricity might be useful.

As prevention and cure are the chief uses of surgeons, it seems proper to make some remarks on these:—First, as to prevention of such distensions or varices, bleeding profusely may be necessary, in order to lessen the quantity of blood in the vessels, when of course there must be less distension; but, as it may not be free from danger to carry it to a great extent, we ought, therefore, to apply to that remedy which is capable of preventing the occurrence of such vascular distension as arises from an obstruction to respiration, caused by convulsion of the respiratory muscles, viz. opium. This article, by its power of preventing and removing spasmodic action, I have dwelt on sufficiently in a former paper; here, I need not add the propriety of delivery; also, that other means, tending to the same end, though of less powers, should not be neglected. As to cure, here the same indications must be kept in view. As this female, from their accounts, seems to see better than she did at first, it may be a question whether she may not ultimately recover?—I should not think she would; her want of vision for the three weeks after her first attack cannot be proved. Her having recovered somewhat, may partly have arisen from having acquired by experience an ability of using the sensible spots of the retina with greater facility than at first. And, perhaps, where the varices cause but slight pressure, the retina may, in time, become so accustomed to the pressure as to recover its action.

I shall conclude this letter with the following case of muscæ volitantes.

About three months since, J. B. æt. 25, of a dark complexion and robust firm fibre, applied on account of an imperfection of vision on the right eye. Said, that five or six weeks ago, he was struck with the boom of the vessel in which he sails; it caused a black eye. About a week or two after, the same eye became red, accompanied with pricking pains, and an inability of bearing the light. This inflammation got well in about a fortnight without using any remedy. For about a week he has found this same eye to be dim, and can now only distinguish light and shadow. When both eyes are equally exposed to light, the right pupil is evidently smaller than the left, and does not dilate so freely on diminishing the light. It dilates, however, in the natural way on shutting the lids of the left eye, but the left pupil is *scarcely at all* affected by shutting the right eye. The natural sympathy, therefore, of the diseased eye with the sound remains, but the sympathy of the sound eye with the diseased one is nearly suspended. In this case, I should be inclined to adopt the depleting plan, but not sufficient opportunity was afforded, as the vessel soon left this port.—I saw him again about three months after, which is only a few days since. The right eye still continues dim, as when I last saw him; the pupils of both eyes have their healthy and natural motions. For about a month, he says, he has remarked from three to six dark fly-like objects dancing before his left eye. He can see small print, or even the smallest dot on paper, as well as ever; and these spectra seem to him to dance round all objects to which he particularly directs his sight. He sees more spectra, and these more distinctly, after working hard; he does not perceive any when his back is turned to the window, but does immediately on looking towards the window, especially when the light is great. The spectra are larger, when viewing distant objects. After a hard day's work, he says there is an aching in the eye-balls. This young man cannot leave the vessel, and, consequently, cannot remain long enough in one place to allow of the necessary treatment. In his case, I should use the depleting plan,—as leeches, &c.; and, perhaps, sometime hence, when the retina becomes accustomed to pressure, these spectra may cease to appear, as in those cases mentioned by Mr. Ware.

My letter is now extended to a much greater length than I expected; but, as I am not well able to abridge it, I can only beg pardon for any intrusion on your readers.

*Padstow; March* 14, 1818.

